# *Paenibacillus allorhizoplanae* sp. nov. from the rhizoplane of a *Zea mays* root

**DOI:** 10.1007/s00203-022-03225-w

**Published:** 2022-09-18

**Authors:** Peter Kämpfer, André Lipski, Lucie Lamothe, Dominique Clermont, Alexis Criscuolo, John A. McInroy, Stefanie P. Glaeser

**Affiliations:** 1 Institut für angewandte Mikrobiologie, Justus-Liebig-Universität Giessen, Heinrich-Buff-Ring 26–32, 35392 Giessen, Germany; 2 Institut für Ernährungs- und Lebensmittelwissenschaften, Lebensmittelmikrobiologie und –hygiene, Rheinische Friedrich-Wilhelms-Universität Bonn, Bonn, Germany; 3 CNRS, Institut Français de Bioinformatique, IFB-Core, UMS 3601, Evry, France; 4 Institut Pasteur, Université de Paris, Bioinformatics and Biostatistics Hub, 75015 Paris, France; 5 Institut Pasteur, Université de Paris, CIP–Collection of Institut Pasteur, 75015 Paris, France; 6 Department of Entomology and Plant Pathology, Auburn University, Alabama, USA

**Keywords:** *Paenibacillus allorhizoplanae*, Taxonomy, *Zea mays*, Endophyte, Plant associated, Genome sequence

## Abstract

A Gram-positive staining, aerobic, endospore-forming bacterial strain, isolated from the rhizosphere of *Zea mays* was studied for its detailed taxonomic allocation. Based on the 16S rRNA gene sequence similarity comparisons, strain JJ-42^ T^ was shown to be a member of the genus *Paenibacillus*, most closely related to the type strain of *Paenibacillus pectinilyticus* (98.8%). The 16S rRNA gene sequence similarity to all other *Paenibacillus* species was below 98.5%. The pairwise average nucleotide identity (ANI) and digital DNA−DNA hybridization (dDDH) values of the JJ-42^ T^ genome assembly against publicly available *Paenibacillus* type strain genomes were below 92% and 47%, respectively. The quinone system of strain JJ-42^ T^ consisted exclusively of menaquinone MK-7. The polar lipid profile consisted of the major components diphosphatidylglycerol, phosphatidylethanolamine, phosphatidylglycerol, three aminophospholipids (APL), and one unidentified lipid. The major fatty acids were iso- and anteiso-branched with the major compound anteiso C_15:0_. Physiological and biochemical characteristics allowed a further phenotypic differentiation of strain JJ-42^ T^ from the most closely related species. Thus, JJ-42^ T^ represents a novel species of the genus *Paenibacillus*, for which the name *Paenibacillus allorhizoplanae* sp. nov. is proposed, with JJ-42^ T^ (= LMG 32089^ T^ = CCM 9085^ T^ = DSM 111786^ T^ = CIP 111891^ T^) as the type strain.

**Supplementary Information:**

The online version contains supplementary material available at 10.1007/s00203-022-03225-w.

## Introduction

The genus *Paenibacillus,* initially proposed by Ash et al. (1993) is now accommodating more than 280 species (https://lpsn.dsmz.de/genus/paenibacillus) isolated from many different sources. It is obvious that species of *Paenibacillus* have been isolated often as endophytes (Carro et al. 2013; Lai et al. 2015; Kittiwongwattana and Thawai 2015; Gao et al. 2015) and from other plant-associated environments as the rhizosphere (Elo et al. 2001; Ma et al. 2007; Kim et al. 2009a, b; Hong et al. 2009; Beneduzi et al. 2010; Zhang et al. 2013; 2015; Wang et al. 2014; Son et al. 2014; Han et al. 2015; Kämpfer et al. 2016, 2017a,b, 2021), seeds (Liu et al. 2015) or the phyllosphere (Rivas et al. 2005, 2006). In general, endospore-forming bacilli, including members of the genus *Paenibacillus*, are of particular interest for their capacity to promote plant growth and form endospores (Grady et al. 2016). The purpose of this study was to analyze strain JJ-42^ T^ in detail for its taxonomic allocation and for the presence of genes associated with plant−growth promotion.

## Materials and methods

### Isolation and culture conditions

Field-grown maize plants (*Zea mays*) grown in Dunbar, Nebraska were manually uprooted a few weeks after planting. Roots, approximately 15 cm in length, were separated from the surrounding soil by vigorous shaking such that only the most tightly adhering soil remained. Bacteria were collected from the root surface by immersing the root in sterile water followed by plating dilutions on Nutrient Agar (Sigma-Aldrich). Strain JJ-42^ T^ was initially isolated at Auburn University in this manner. Subsequent cultivation of JJ-42^ T^ was performed on tryptone soy agar, TSA (Oxoid) at 28 °C for 24 h.

### Molecular characterization

#### 16S rRNA gene phylogeny

For a first phylogenetic placement the 16S rRNA gene of strain JJ-42^ T^ was PCR amplified with primer system Eub9f (5´-GAGTTTGATCMTGGCTCAG-3´) and Eub1492R (5´-ACGGYTACCTTGTTACGACTT-3´) (Lane 1991) and sequenced with the Sanger dideoxy sequencing technology using primers Eub9f and E786F (5´-GATTAGATACCCTGGTAG-3´), respectively, as described previously (Schauss et al. 2015). The sequence was manually processed and corrected based on the electropherograms using MEGA11 (Tamura et al. 2021).

A first phylogenetic assignment was performed by BLAST analysis against the EzBioCloud 16S rRNA gene sequence database, including all type strain 16S rRNA gene sequences and BLASTN BLAST + 2.13.0 (reduced to sequences of type material) of the NCBI (https://blast.ncbi.nlm.nih.gov/). The 16S rRNA gene sequences of strain JJ-42^ T^ and nonincluded-related type strains were subsequently added to LTP_2020 database of the “All-Species Living Tree'' Project (LTP; Yarza et al. 2008) using ARB release 5.2 (Ludwig et al. 2004). The updated database version LTP_04_2021 (released in September 2021) was used. The imported 16S rRNA gene sequences were aligned in the alignment explorer using the pt server generated for the respective database. The alignment was checked manually before subsequent analyses. The sequences were added to the database tree tree_Ltp_all_04_21 containing all type strain 16S rRNA gene sequences of species described until May 2021 using the parsimony quick ad marked tool of ARB and the gap95_q0_to_q5 filter.

All strains of the resulting cluster and the type strain of the type species *Paenibacillus polymyxa* and of two additional species, *Paenibacillus peoriae* and *Paenibacillus kribbensis*, which clustered with the type species, were included in the analysis. Type strains of two *Cohnella* species*, Cohnella thermotolerans* and *Cohnella xylanilytica,* were used as outgroup. The genus *Cohnella* is a monophyletic genus related to the paraphyletic genus *Paenibacillus*. A maximum-likelihood tree was calculated with RAxML v7.04 (Stamatakis 2006), GTR-GAMMA and rapid bootstrap analysis and a maximum-parsimony tree with DNAPARS v 3.6 (Felsenstein 2005). Both trees were calculated with 100 re-samplings (bootstrap analysis; Felsenstein 1985) and based on the 16S rRNA gene sequences between gene termini 95 to 1475 (*Escherichia coli* numbering, Brosius et al. 1978).

#### Genomic features

The whole-genome sequencing of strains JJ-42^ T^ was carried out with a NextSeq500 instrument using the Nextera XT DNA library preparation kit (Illumina) and a 2 × 150 bp paired-end protocol, yielding 3,876,943 read pairs (140 × average sequencing depth, 356 bp average insert size). Read processing, genome assembly and scaffolding, and gene prediction and annotation were performed using fq2dna v21.06 (https://gitlab.pasteur.fr/GIPhy/fq2dna).

Different plant−beneficial function contributing (PBFC) genes (as listed in Cherif-Silini et al. 2019) were searched using tblastn against the draft genome of JJ-42^ T^, assessing the presence of different putative PBFC genes related to different features.

Pairwise average nucleotide and amino acid identity (ANI and AAI, respectively) values were computed using OGRI_B (https://gitlab.pasteur.fr/GIPhy/OGRI) between the draft genome of JJ-42^ T^ and every *Paenibacillus* type strain genome selected for the 16S rRNA gene sequence based phylogenetic analysis (when publicly available).

### Physiology and chemotaxonomy

Detailed phenotypic characterization of strain JJ-42^ T^ was performed in comparison to *P. pectinilyticus* KCTC 13222^ T^. Cell morphology and motility were observed under a Zeiss light microscope at a magnification of 1000, using cells that had been grown for 3 days at 28 °C on TSA (Oxoid). Gram staining was performed by the modified Hucker method according to Gerhardt et al. (1994). Oxidase activity was tested with an oxidase reagent text kit following the instructions of the manufacturer (bioMérieux, France). The KOH test for the determination of spores was carried out according to Moaledj (1986). The growth was tested on different agar media, including R2A (Oxoid), nutrient broth agar (NB, Oxoid), tryptic soy agar (TSA, Becton Dickinson), malt agar (Merck), PYE [0.3% (w/v) yeast extract, and 0.3% (w/v) casein peptone, respectively, 15 g agar L^−1^, pH 7.2], CASO agar (Carl Roth), K7 [0.1% (w/v) of yeast extract, peptone, and glucose, 15 g L^−1^ agar, pH 6.8], medium 65 (M65, according to DSMZ), DEV agar (DEV, Merck), Nutrient agar (NA, Becton Dickinson), Luria–Bertani agar (LB, Sigma-Aldrich), Marine agar 2216 (MA, Becton Dickinson), Columbia agar with sheep blood (Oxoid), and MacConkey agar (Oxoid). The growth was evaluated after 48 h incubation at 28 °C. Temperature-dependent growth was determined on Columbia agar with sheep blood at 4, 10, 15, 20, 25, 28, 30, 36, 45, 50, and 55 °C. pH and salinity-dependent growth was tested in R2A broth incubated at 28 °C. The pH was adjusted to pH 4.5 to 10.5 (1 pH unit intervals incrementing) using HCl and NaOH. For salinity-dependent growth, 1 to 8% (w/v) NaCl was added (in 1% intervals incrementing). The growth was monitored after 72 h of incubation.

Further physiological characterization of the strains was performed with the API20NE and APIZYM test systems according to the instruction of the manufacturer (bioMérieux) and with the methods described by Kämpfer et al. (1991) and Kämpfer (1990). All tests were incubated at 28 °C.

For analyses of quinones and polar lipids cells were grown in half-concentrated nutrient broth at 25 °C for 7 days. Quinones were extracted as described by Minnikin et al. (1984) and by Wiertz et al. (2013), and analyzed with an Agilent 1260 infinity HPLC system. Polar lipids were extracted and analyzed by thin-layer chromatography (TLC) according to Minnikin et al. (1984). Molybdophosphoric acid was used for visualization of all polar lipids. Aminolipids were stained by ninhydrin, molybdenum blue reagent for the detection of phospholipids, and α-naphthol reagent for the detection of sugar-containing lipids.

Fatty acid analysis was performed in parallel with JJ-42^ T^ and *P. pectinilyticus* KCTC 13222^ T^. The fatty acids were extracted and analyzed as described by Kämpfer & Kroppenstedt (1996). Strains were grown under identical conditions (TS-medium after 72 h incubation at 28 °C) and the cells for extractions were taken from colonies of the same size. Fatty acids were identified with the Sherlock version 2.11, TSBA40 Rev. 4.1.

## Results and discussion

### Molecular and genome characteristics

The final corrected 16S rRNA gene sequence of strain JJ-42^ T^ (OP023150) had a size of 1,466 nt and spanned gene termini 8 to 1,475 (numbering according to the *Escherichia coli rrnB* sequence published by Brosius et al. 1978).

**Fig. 1:**
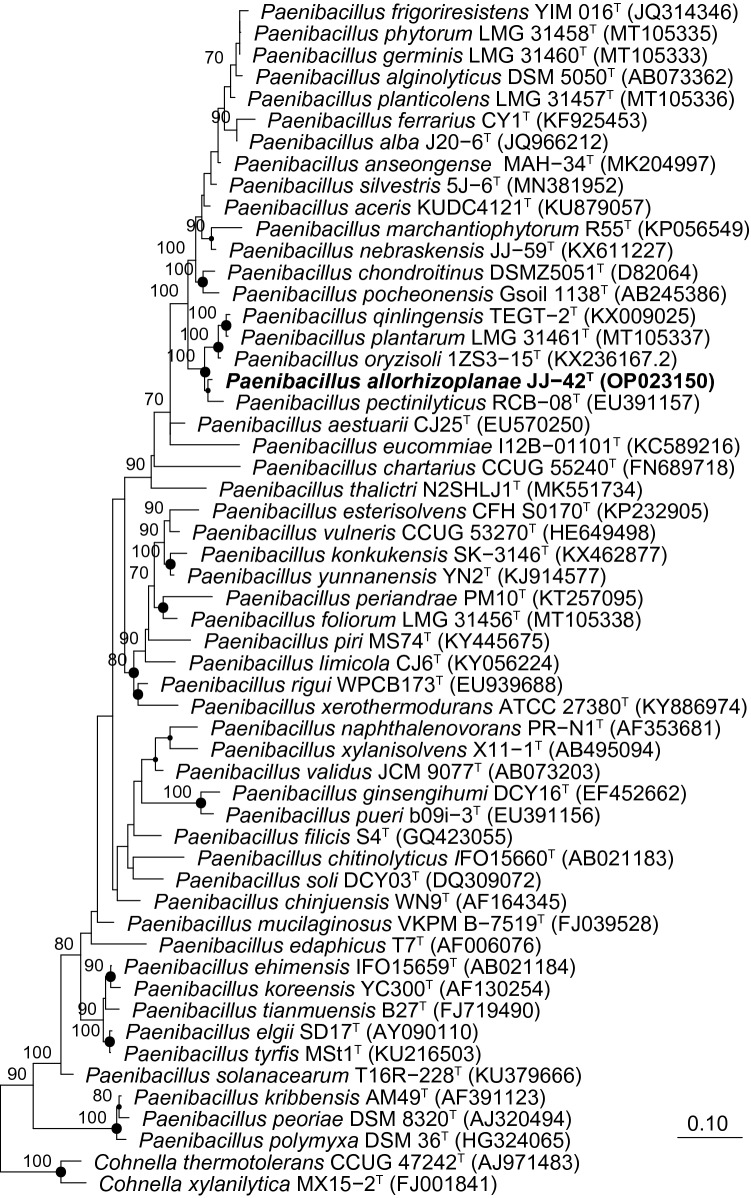
Maximum-likelihood tree showing the phylogenetic position of strain JJ-42^ T^ among the closest related *Paenibacillus* species. The tree was generated in ARB using RAxML (GTR-GAMMA, rapid bootstrap analysis) and based on the 16S rRNA gene sequences between positions 95–1475 according to *E. coli* numbering (Brosius et al. 1978). GenBank/EMBL/DDBJ accession numbers are given in parentheses. Numbers at branch nodes refer to bootstrap values > 70% (100 replicates). Circle marks nodes that were also present in the maximum-parsimony tree. Larger circles were supported by high bootstrap values in the maximum-parsimony tree. Type strains of *Cohnella* species were used as outgroup. Bar, 0.1 substitutions per nucleotide position

Strain JJ-42^ T^ was placed within the cluster of *Paenibacillus* type strains by BLAST analyses. Strain JJ-42^ T^ showed the highest 16S rRNA gene sequence similarity to the type strain of *Paenibacillus pectinilyticus* (98.8%), followed by the type strains of *Paenibacillus qinlingensis* and *Paenibacillus oryzisoli* (both 98.4%). Sequence similarities to all other type strains were below 98%. Independent of the applied treeing method, strain JJ-42^ T^ formed a stable cluster (100% bootstrap support) with the type strains of *P. pectinilyticus*, *P. qinlingensis*, *P. oryzisoli*, and *P. plantarum* (Fig. 1).

The resulting draft genome was made of 8,162,391 bps on 78 contigs (N50, 232,911) with G + C content of 45.07 mol%. A total of 7,004 coding sequences and 103 tRNA was inferred. Genome sequence authenticity was assessed by aligning the 16S rRNA segment derived from Sanger sequencing (OP023150) against the de novo assembly (CAKMMW00000000) using blastn, leading to > 99.4% pairwise sequence similarity.

**Fig. 2:**
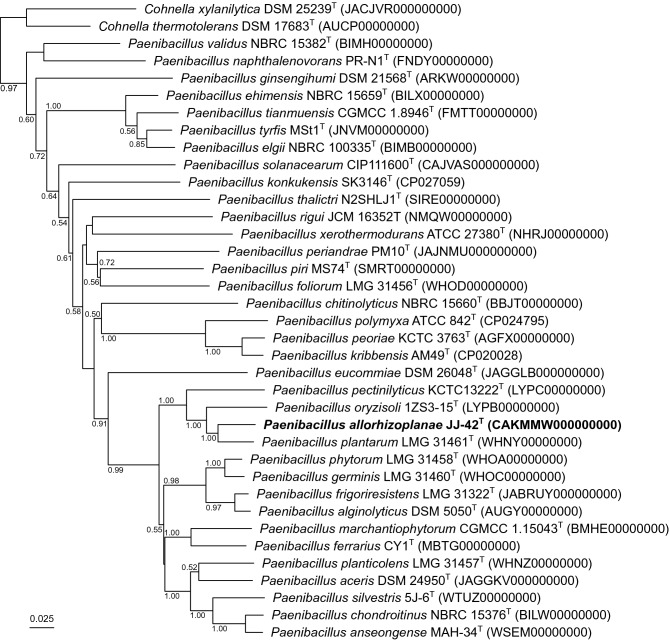
Whole-genome-based tree showing the phylogenetic placement of strain JJ-42^ T^ among type strains of closely related *Paenibacillus* species. This minimum evolution tree was inferred using JolyTree (https://gitlab.pasteur.fr/GIPhy/JolyTree). Two publicly available *Cohnella* type strain genomes were used as an outgroup. The genome sequence accession is specified between parentheses next after each taxon name. Branch supports were assessed by the rate of elementary quartets, as estimated by JolyTree (only supports > 0.5 were specified). Bar, 0.025 nucleotide substitutions per site

ANI and AAI values are reported in Table S1, together with the associated digital DNA−DNA hybridization (dDDH) values (formula 2; https://ggdc.dsmz.de/). All these estimated pairwise similarity values are far below the commonly admitted species delineation cutoffs (ANI, 95%; AAI, 95%; dDDH, 70%). A phylogenomic classification of these genome sequences was also inferred using JolyTree v2.0 (Criscuolo 2019, 2020), such a tree confirming that strain JJ-42^ T^ formed a stable cluster with the type strains of *P. plantarum*, *P. oryzisoli*, and *P. pectinilyticus* (Fig. 2).

Different plant−beneficial function contributing (PBFC) genes (as listed in Cherif-Silini et al. 2019) were searched using tblastn against the draft genome of JJ-42^ T^, assessing the presence of different putative PBFC genes related to plant−root colonization (Table S2), nutrient acquisition (Table S3), growth-promoting traits (Table S4), oxidative stress protection (Table S5), drug and heavy metal resistance (Table S6), disease resistance (Table S7), and degradation of aromatic compounds (Table S8). Finally, seven biosynthetic gene clusters for secondary metabolites were found using antiSMASH (Blin et al. 2021) bacterial version (Table S9).

### Phenotype

The results of the physiological characterization, performed using methods described previously (Kämpfer 1990; Kämpfer et al. 1991), are given in Table 1 and in the species description.

**Table 1:** Differential characteristics of strain JJ-42^ T^ and phylogenetically related species of the genus *Paenibacillus*

Characteristic	1	2	3	4	5	6	7
Source	Rhizoplane of a *Zea mays root*	Gut of *Diestrammena apicalis* (Korea)	Root of *Sinopodophyllum hexandrum* (China)	Soil (China)	Soil (China)	Soil (U.S.A)	Soil (U.S.A)
Nitrate reduction	–	+	+	–	–	+	+
H_2_S production	–	–	–	–	–	–	+
Urease	–	+	–	+	–	–	–
Growth at/in:							
4 °C	–	–	–	+	+	–	–
37 °C	+	+	–	+	+	+	+
1% NaCl	+	–	+	–	+	–	+
Hydrolysis of:							
Aesculin	+	+	–	+	+	+	+
Starch	–	–	–	+	–	–	–
Assimilation of (API 20NE):							
*N*-Acetylglucosamine	+ *	–	+	+	–	–	+
L-Arabinose	–	–	–	+	–	–	+
D-Glucose	–	–	+	+	–	–	+
Maltose	+ *	–	+	+	+	–	+
D-Mannitol	–	–	–	+	–	–	+
D-Mannose	–	–	–	+	–	–	-
Potassium gluconate	+ *	–	–	+	–	–	+
Enzyme activities (API ZYM)							
Acid phosphatase	–	–	–	+	+	–	+
*α*-Chymotrypsin	–	–	–	+	–	+	–
Cystine arylamidase	–	+	–	+	w	+	–
*α* -Galactosidase	–	–	–	+	–	+	–
*α* Glucosidase	–	–	w	+	+	-	w
*β*-Glucosidase	+	+	+	+	–	–	–
Trypsin	–	–	–	+	–	–	–
Valine arylamidase	+	–	–	+	+	–	–

Strains: 1, JJ-42^ T^; 2, *Paenibacillus pectinilyticus* KCTC 13222^ T^; 3, *Paenibacillus qinlingensis* TEGT-2^ T^; 4, *Paenibacillus frigoriresistens* CCTCC AB 2011150^ T^; 5, *Paenibacillus ferrarius* CCTCC AB 2013369^ T^; 6, *Paenibacillus alginolyticus* NBRC 15375^ T^; 7, *Paenibacillus chondroitinus* DSM 5051^ T^. Data for taxa 1 and 2 from this study; all other data are from Xin et al. (2017). + , Positive; –, negative; w, weakly positive

^*^After 48 h of incubation

The polar lipid profile consisted of diphosphatidylglycerol, phosphatidylethanolamine, phosphatidylglycerol, three unidentified aminophospholipids, and one unidentified polar lipid **(**Fig. S1). The quinone system of strain JJ-42^T^ consisted exclusively of menaquinone MK-7. The presence of menaquinone MK-7 as well as the polar lipid profile are in agreement with the characteristics of other species of the genus *Paenibacillus*.

The fatty acids comprised mainly iso- and anteiso-branched fatty acids and the fatty acid profile was very similar to the most closely related *Paenibacillus* species. The detailed fatty acid profile obtained from cells grown is shown in Table S10.

## Conclusion

Based on the summary of genotypic and phenotypic, we describe a novel species of the genus *Paenibacillus*, for which the name *Paenibacillus allorhizoplanae* is proposed with JJ-42^ T^ as proposed type strain.

### Description of *Paenibacillus allorhizoplanae* sp. nov.

*Paenibacillus allorhizoplanae ***(**Gr. masc. adj. *allos*, other; N.L. gen. n. *rhizoplanae*, a specific epithet ('of the rhizoplane'); N.L. gen. n. *allorhizoplanae*, related, but distinct from *Paenibacillus rhizoplanae*).

Cells (with rounded ends) stain Gram-positive. No chains or filaments could be observed after growth on TSA at 28 °C for 48 h. Cells were 2.0–3.0 µm in length and 0.8–1.0 µm in width) and showed no motility. Oval endospores are formed in a subterminal position in swollen sporangia. No other cell inclusions could be detected. Colonies grown on tryptone soy agar (after 48 h of incubation on TSA) are circular, convex, and beige with a shiny appearance and an average diameter of 2–3 mm. The growth at 28 °C on R2A (Oxoid), NB (Oxoid), TSA, Malt agar (Merck), PYE, CASO agar (Carl Roth), K7, M65, DEV agar (DEV, Merck), NA (Becton Dickinson), LB (Sigma-Aldrich), and Columbia agar with sheep blood (Oxoid). No growth on MA (Becton Dickinson) and MacConkey agar (Oxoid).

Good growth on blood agar between 25 and 45 °C (optimal growth at 36 °C), growth occurred between 15 and 45 °C, but not at 4, 10, 50 °C, and above. Optimal pH for growth in R2A broth at 28 °C is pH 7–8; growth occurs between pH 4.5 and 10.5. Optimal growth in R2A broth at 28 °C without and in the presence of 1% NaCl, growth occurs between 0 and 3% NaCl, but not at 4% or above. Both, pH- and salinity-dependent growth tested in R2A broth at 28 °C. Tests for catalase and oxidase activities are negative. According to API 20 NE, positive for the activity of ß-glucosidase (esculin hydrolysis), PNPG beta-galactosidase, and the assimilation of potassium gluconate, and negative for the activity of nitrate reduction, indol production, D-glucose fermentation, arginine dihydrolase, urease, gelatin hydrolysis, and the assimilation of D-glucose, L-arabinose, D-mannose, D-mannitol, N-acetylglucosamine, D-maltose, capric acid, adipic acid, malic acid, trisodium citrate, and phenylacetic acid.

According to API ZYM, positive for leucine arylamidase, acid phosphatase, naphtol-AS-BI-phosphohydrolase, and *β*-galactosidase, and negative for alkaline phosphatase, esterase (C4), esterase lipase (C8), lipase (C14), valine arylamidase, cystine arylamidase, trypsin, *α*-chymotrypsin, *α-*galactosidase, *β*-glucuronidase, *α*-glucosidase, *β*-glucosidase, N-acetyl-*β*-glucosaminidase, *α*-mannosidase, and *α*-fucosidase.

Some sugars or sugar-related compounds were utilized: L-arabinose (weak), D-galactose, D-gluconate, glucose (weak), D-melibiose, ribose, D-trehalose, and D-maltitol are utilized as sole sources of carbon.

Arbutin, D-cellobiose, D-fructose, i-inositol, D-mannose, D-maltose, salicin, sucrose, D-sorbitol, D-xylose, acetate, N-acetyl-D-glucosamine, cis-aconitate, trans-aconitate, adipate, D-adonitol, 4-aminobutyrate, azelate, citrate, itaconate, malate, mesaconate, 2-oxoglutarate, propionate, putrescine, pyruvate and L-rhamnose are not utilized as sole carbon source.

The quinone system contains only menaquinone MK-7. The polar lipid profile is composed of the major lipids diphosphatidylglycerol, phosphatidylglycerol, phosphatidylethanolamine, three unidentified aminophospholipids (APL1-3) and one unidentified polar lipid (L).

The major fatty acids are anteiso C_15:0_ and iso C_16:0_. The genomic DNA G + C content is 45.07 mol% (based on the genome sequence).

The type stain JJ-42^ T^ **(**= LMG 32089^ T^ = CCM 9085^ T^ = DSM 111786^ T^ = CIP 111891^ T^) was isolated from the root surface of a field-grown corn plant in Dunbar, Nebraska USA.

The genome sequence of the type strain is available under accession number CAKMMW00000000 and the 16S rRNA gene sequence under OP023150.

## Supplementary Information

Below is the link to the electronic supplementary material.

Supplementary file1 (PDF 660 KB)

## Data Availability

The new generated sequences were uploaded to the GenBank database at the National Center for Biotechnology Information (NCBI) and are available. The complete genome sequence of strain JJ-42^ T^ has been deposited under the GenBank/EMBL/DDBJ accession numbers CAKMMW000000000 and the 16S rRNA gene sequence under OP023150.
