# Supplementary material for: Paenibacillus allorhizoplanae sp. nov. from the rhizoplane of a Zea mays root
Source: Arch Microbiol. 2022 Sep 18;204(10):630. doi: 10.1007/s00203-022-03225-w (PMC9482572; doi:10.1007/s00203-022-03225-w)

Archives of Microbiology

Paenibacillus allorhizoplanae sp. nov. from the rhizoplane of a *Zea mays* root

Peter Kämpfer [1,*], Andre Lipski [2], Lucie Lamothe [3,4], Dominique Clermont [5], Alexis Criscuolo [4], John A. McInroy [6], and Stefanie P. Glaeser [1]

[1] Institut für Angewandte Mikrobiologie, Universität Giessen, Germany

[2] Institut für Ernährungs- und Lebensmittelwissenschaften, Lebensmittelmikrobiologie und –hygiene, Rheinische Friedrich-Wilhelms-Universität Bonn, Germany

[3] CNRS, Institut Français de Bioinformatique, IFB-core, UMS 3601, Evry, France

[4] Institut Pasteur, Université de Paris, Bioinformatics and Biostatistics Hub, F-75015 Paris, France

[5] Institut Pasteur, Université de Paris, CIP - Collection of Institut Pasteur, F-75015 Paris, France

[6] Department of Entomology and Plant Pathology, Auburn University, Alabama, USA

[*] Corresponding author (peter.kaempfer@umwelt.uni-giessen.de)

Supplementary Information

**Table S1**	Overall genome relatedness indices
**Tables S2-S8**	Putative plant-beneficial function contributing genes
**Table S9**	Seven gene clusters for secondary metabolites predicted by antiSMASH
**Table S10**	Cellular fatty acid profiles
**Figure S1**	Polar lipid profile

Table S1. Overall genome relatedness indices between strain JJ-42^T and next related *Paenibacillus* type strains

type strain	genome accession	%ANI [CI]	%AAI [CI]	%dDDH [CI]
P. plantarum LMG 31461^T	WHNY00000000	91.46 [91.29-91.61]	94.08 [93.84-94.31]	46.30 [43.7-48.9]
P. oryzisoli 1ZS3-15^T	LYPB00000000	87.03 [86.85-87.19]	91.07 [90.80-91.31]	33.40 [31.0-36.0]
P. pectinilyticus KCTC13222^T	LYPC00000000	81.05 [80.87-81.24]	86.28 [85.96-86.61]	24.50 [22.2-26.9]
P. germinis LMG 31460^T	WHOC00000000	77.27 [77.09-77.47]	80.51 [80.12-80.86]	22.10 [19.8-24.5]
P. phytorum LMG 31458^T	WHOA00000000	77.15 [76.94-77.33]	80.42 [80.07-80.79]	21.90 [19.6-24.3]
P. frigoriresistens LMG 31322^T	JABRUY000000000	77.03 [76.78-77.23]	80.62 [80.25-81.04]	21.70 [19.4-24.1]
P. alginolyticus DSM 5050^T	AUGY00000000	76.92 [76.70-77.16]	80.56 [80.11-81.02]	21.50 [19.3-24.0]
P. marchantiophytorum CGMCC 1.15043^T	BMHE00000000	76.09 [75.84-76.31]	79.64 [79.23-80.02]	20.90 [18.7-23.4]
P. aceris DSM 24950^T	JAGGKV000000000	75.89 [75.67-76.13]	79.45 [79.01-79.85]	20.70 [18.5-23.1]
P. ferrarius CY1^T	MBTG00000000	75.87 [75.63-76.10]	79.17 [78.75-79.57]	20.80 [18.6-23.3]
P. planticolens LMG 31457^T	WHNZ00000000	75.79 [75.55-75.99]	79.34 [78.89-79.75]	20.30 [18.1-22.7]
P. chondroitinus NBRC 15376^T	BILW00000000	75.44 [75.20-75.67]	78.89 [78.52-79.27]	20.20 [18.0-22.6]
P. silvestris 5J-6^T	WTUZ00000000	75.39 [75.14-75.61]	78.81 [78.40-79.13]	20.30 [18.0-22.7]
P. anseongense MAH-34^T	WSEM00000000	75.38 [75.13-75.59]	78.61 [78.31-78.99]	20.30 [18.1-22.7]
P. eucommiae DSM 26048^T	JAGGLB000000000	69.83 [69.55-70.06]	68.86 [68.39-69.33]	19.70 [17.5-22.1]
P. rigui JCM 16352^T	NMQW00000000	68.80 [68.58-69.05]	66.69 [66.16-67.13]	20.50 [18.2-22.9]
P. foliorum LMG 31456^T	WHOD00000000	68.79 [68.49-69.01]	66.45 [65.97-66.90]	21.50 [19.3-23.9]
P. periandrae PM10^T	JAJNMU000000000	68.60 [68.38-68.88]	65.97 [65.56-66.40]	21.30 [19.1-23.7]
P. piri MS74^T	SMRT00000000	68.50 [68.24-68.75]	66.26 [65.74-66.69]	20.60 [18.4-23.1]
P. chitinolyticus NBRC 15660^T	BBJT00000000	68.46 [68.18-68.68]	65.97 [65.50-66.44]	19.60 [17.4-22.0]
P. tianmuensis CGMCC 1.8946^T	FMTT00000000	68.40 [68.10-68.66]	66.19 [65.65-66.72]	21.30 [19.1-23.8]
P. naphthalenovorans PR-N1^T	FNDY00000000	68.32 [68.03-68.56]	66.36 [65.80-66.89]	20.70 [18.5-23.1]
P. solanacearum CIP111600^T	CAJVAS000000000	68.32 [68.09-68.53]	65.59 [65.18-66.03]	21.40 [19.2-23.9]
P. konkukensis SK3146^T	CP027059	68.25 [68.02-68.50]	66.17 [65.71-66.57]	25.80 [23.5-28.3]

Table S1. (cont.)

type strain	genome accession	%ANI [CI]	%AAI [CI]	%dDDH [CI]
P. validus NBRC 15382^{T}	BIMH00000000	68.23 [67.95-68.49]	66.37 [65.83-66.88]	19.20 [17.0-21.5]
P. xerothermodurans ATCC 27380^{T}	NHRJ00000000	68.18 [67.89-68.44]	66.44 [65.95-66.99]	21.50 [19.3-23.9]
P. thalictri N2SHLJ1^{T}	SIRE00000000	68.04 [67.82-68.26]	65.82 [65.30-66.30]	20.60 [18.3-23.0]
P. tyrfis MSt1^{T}	JNVM00000000	68.00 [67.71-68.25]	65.55 [65.01-65.97]	20.00 [17.8-22.4]
P. ehimensis NBRC 15659^{T}	BILX00000000	67.98 [67.76-68.19]	65.72 [65.23-66.21]	19.10 [16.9-21.4]
P. elgii NBRC 100335^{T}	BIMB00000000	67.88 [67.63-68.13]	65.68 [65.07-66.09]	18.70 [16.6-21.1]
P. ginsengihumi DSM 21568^{T}	ARKW00000000	67.53 [67.32-67.77]	65.23 [64.67-65.68]	20.60 [18.4-23.1]
P. kribbensis AM49^{T}	CP020028	67.22 [66.90-67.51]	62.57 [62.02-63.09]	28.80 [26.4-31.3]
P. polymyxa ATCC 842^{T}	CP024795	67.13 [66.80-67.43]	62.33 [61.81-62.79]	33.10 [30.7-35.6]
P. peoriae KCTC 3763^{T}	AGFX00000000	67.04 [66.73-67.33]	62.34 [61.88-62.95]	21.30 [19.1-23.7]

Table S2. Genes of strain JJ-42^T potentially related with root colonization/growth promoting factors

accession	gene	annotated product	putative function
CAH1199518	swrC_1	Swarming motility protein	Swarming motility
CAH1203149	flgB	Flagellar basal body rod protein	
CAH1203152	flgC		
CAH1203179	-	-	
CAH1203184	flgG_1	Flagellar basal-body rod protein	
CAH1203217	flhB	Flagellar biosynthesis protein	
CAH1203220	flhA		
CAH1203222	ftsY_2	Signal recognition particle receptor	
CAH1215848	flaB3	Flagellar filament 30.7 kDa core protein	
CAH1215854	-	-	
CAH1215859	yvyG	-	
CAH1215864	-	-	
CAH1215982	flgG_2		
CAH1215984	flgG_3		
CAH1218165	swrC_2		
CAH1229913	swrC_3		
CAH1197206	cheR_1	Chemotaxis protein methyltransferase	Chemotaxis ability
CAH1200060	motB_1	Motility protein B	
CAH1200063	pomA_1	Chemotaxis protein	
CAH1202078	cheA_1	Chemotaxis protein	
CAH1202079	cheW_1	Chemotaxis protein	
CAH1203155	fliE	Flagellar hook-basal body complex protein	
CAH1203158	-	-	
CAH1203161	fliG	Flagellar motor switch protein	
CAH1203164	-	-	
CAH1203167	yscN	putative ATP synthase	
CAH1203170	-	-	
CAH1203175	-	-	
CAH1203190	-	-	
CAH1203194	fliM	Flagellar motor switch protein	
CAH1203197	-	-	
CAH1203207	fliP	Flagellar biosynthetic protein	
CAH1203211	fliQ	Flagellar biosynthetic protein	
CAH1203214	fliR	Flagellar biosynthetic protein	
CAH1203231	cheA_1		
CAH1203234	cheW_2		
CAH1203237	cheC	CheY-P phosphatase	
CAH1215641	motB_2		
CAH1215748	fliS	Flagellar secretion chaperone	
CAH1215752	-	-	
CAH1197382	xerD	Tyrosine recombinase	Rhizosphere colonization
CAH1203604	xerC_1		
CAH1205308	xerC_2		
CAH1208296	xerC_3		
CAH1207625	epsL_1	putative sugar transferase	Exopolysaccharide biosynthesis
CAH1207629	ywqD	Tyrosine-protein kinase	
CAH1207631	ywqC_1	putative capsular polysaccharide biosynthesis	
CAH1207677	epsN	Putative pyridoxal phosphate-dependent aminotransferase	
CAH1207681	epsM_1	Putative acetyltransferase	
CAH1207684	epsL_2		
CAH1207695	epsG	Transmembrane protein	
CAH1207702	epsF_3	Putative glycosyltransferase	
CAH1207706	epsD	Putative glycosyltransferase	
CAH1207714	pglF_1	UDP-N-acetyl-alpha-D-glucosamine C6 dehydratase	
CAH1207753	-	-	
CAH1207804	-	-	
CAH1207832	yveL_1	Putative tyrosine-protein kinase	
CAH1207837	ywqC_2		
CAH1208377	wcaJ	UDP-glucose:undecaprenyl-phosphate glucose-1-phosphate transferase	
CAH1211470	capD	UDP-glucose 4-epimerase	
CAH1211480	pglC	Undecaprenyl phosphate N,N'-diacetylbacillosamine 1-phosphate transferase	
CAH1211485	pglF_2	UDP-N-acetyl-alpha-D-glucosamine C6 dehydratase	
CAH1211491	yveL_2		
CAH1211497	ywqC_3		
CAH1215791	epsM_2		
CAH1230001	-	-	

Table S3. Genes of strain JJ-42^T potentially related with nutrient acquisition

accession	gene	annotated product	putative function
CAH1209554	ureD	Urease accessory protein	Urea utilization
CAH1209557	ureG		
CAH1209560	ureF		
CAH1209563	ureC	Urease subunit alpha	
CAH1209566	ureB	Urease subunit beta	
CAH1209569	ureA	Urease subunit gamma	

Table S4. Genes of strain JJ-42^T potentially related with plant growth-promoting traits

accession	gene	annotated product	putative function
CAH1195479	aldHT_1	Aldehyde dehydrogenase, thermostable	Auxin biosynthesis
CAH1200235	aldHT_2		
CAH1203616	tpa	Taurine--pyruvate aminotransferase	
CAH1221401	aldHT_3		
CAH1229998	aldH1	4,4'-diaponeurosporen-aldehyde dehydrogenase	
CAH1230169	ysnE	putative N-acetyltransferase YsnE	
CAH1231088	betB	NAD/NADP-dependent betaine aldehyde dehydrogenase	
CAH1196763	miaA	tRNA dimethylallyltransferase	Cytokinin biosynthesis
CAH1203503	miaB	tRNA-2-methylthio-N(6)-dimethylallyladenosine synthase	
CAH1195168	nasE_1	Assimilatory nitrite reductase [NAD(P)H] small subunit	Nitric oxide production
CAH1195172	nasD_2	Nitrite reductase [NAD(P)H]	
CAH1196881	speD	S-adenosylmethionine decarboxylase proenzyme	Polyamines biosynthesis
CAH1204800	speA_1	Arginine decarboxylase	
CAH1215910	metK	S-adenosylmethionine synthase	
CAH1218472	speE_1	Polyamine aminopropyltransferase	
CAH1228937	speA_2		

Table S5. Genes of strain JJ-42^T potentially related with plant protection from oxidative stress

accession	gene	annotated product	putative function
CAH1192185	katG	Catalase-peroxidase	Antioxidant enzymes
CAH1196255	bsaA_1	Glutathione peroxidase	
CAH1199602	tpx_1	Thiol peroxidase	
CAH1200308	ydbD_1	putative manganese catalase	
CAH1200361	ohrR	Organic hydroperoxide resistance transcriptional regulator	
CAH1206062	tpx_2		
CAH1206161	hmp	Flavohemoprotein	
CAH1206522	sodA	Superoxide dismutase [Mn]	
CAH1206583	bcp	Putative peroxiredoxin	
CAH1222441	ohrA	Organic hydroperoxide resistance protein	
CAH1223652	ywrD	Glutathione hydrolase-like YwrD proenzyme	
CAH1228009	katA_1	Vegetative catalase	
CAH1230895	ydbD_2		
CAH1231724	-	Alkyl hydroperoxide reductase C	
CAH1232038	katA_2	Catalase	

Table S6. Genes of strain JJ-42^{T} potentially related with drug and heavy metal resistances

accession	gene	annotated product	putative function
CAH1217504	fosB	Metallothiol transferase	Fosfomycin resistance
CAH2716734	aadK	Aminoglycoside 6-adenylyltransferase	Aminoglycoside 6-adenylyltransferase
CAH1192657<br>CAH1200302<br>CAH1205994<br>CAH1206429<br>CAH1223293<br>CAH1223303<br>CAH1223307	-<br>-<br>-<br>-<br>arsC<br>-<br>arsR	-<br>-<br>-<br>-<br>Arsenate reductase<br>-<br>Arsenical resistance operon repressor	Arsenic detoxification
CAH1212180<br>CAH1226351<br>CAH1226355<br>CAH1226364	ycnJ<br>copA<br>copZ<br>csoR	Copper transport protein<br>Copper-exporting P-type ATPase<br>Copper chaperone<br>Copper-sensing transcriptional repressor	Copper resistance
CAH1196705	-	-	Aluminium resistance
CAH1200303<br>CAH1209260	czcD_1<br>czcD_2	Cadmium, cobalt and zinc/H(+)-K(+) antiporter	Cation Diffusion Facilitator Transporters
CAH1197393<br>CAH1212190	fur<br>nikR	Ferric uptake regulation<br>nickel-responsive transcriptional regulator	Metalloregulators
CAH1208339<br>CAH1208347	ndoA<br>ndoAI	Endoribonuclease EndoA<br>Antitoxin EndoAI	Toxin-Antitoxin Module response to stress

Table S7. Genes of strain JJ-42^{T} potentially related with disease resistance

accession	gene	annotated product	putative function
CAH1208473<br>CAH1209612<br>CAH1218916<br>CAH1225259<br>CAH1225264<br>CAH1225269<br>CAH1227356	ilvG<br>ilvD<br>ilvA<br>ilvB<br>ilvH<br>ilvC<br>ilvB2	Acetolactate synthase isozyme 2 large subunit<br>Dihydroxy-acid dehydratase<br>L-threonine dehydratase biosynthetic<br>Acetolactate synthase large subunit<br>Acetolactate synthase small subunit<br>Ketol-acid reductoisomerase (NADP(+))<br>Putative acetolactate synthase large subunit	Acetoine and 2,3-butandiol biosynthesis
CAH1200166<br>CAH1229980	davT<br>gabR	5-aminovalerate aminotransferase<br>HTH-type transcriptional regulatory protein	GABA biosynthesis

Table S8. Genes of strain JJ-42^{T} potentially related with degradation of aromatic compounds

accession	gene	annotated product	putative function
CAH1199532<br>CAH1203858<br>CAH1206167<br>CAH1227974<br>CAH1228439<br>CAH1228442<br>CAH1228445<br>CAH1232511	yodC<br>azoR2_1<br>azoR2_2<br>mhqR_1<br>mhqD<br>mhqO<br>mhqR_2<br>azoR2_3	Putative NAD(P)H nitroreductase<br>FMN-dependent NADH-azoreductase 2<br><br>HTH-type transcriptional regulator<br>Putative hydrolase<br>Putative ring-cleaving dioxygenase	2-Methylhydroquinone and catechol resistance

Table S9. Biosynthetic gene clusters of strain JJ-42^T for secondary metabolites

accession	gene	annotated product	type
CAH1191526	yteP_1	putative multiple-sugar transport system permease	Type III Polyketide synthase
CAH1191530	-	-	
CAH1191536	rssB_1	Regulator of RpoS	
CAH1191541	-	-	
CAH1191546	yfmO	Multidrug efflux protein	
CAH1191551	yfmP	HTH-type transcriptional regulator	
CAH1191556	yfbR_1	5'-deoxynucleotidase	
CAH1191558	rcsC_1	Sensor histidine kinase	
CAH1191562	rhaR_2	HTH-type transcriptional activator	
CAH1191566	-	-	
CAH1191568	mmpL3	Trehalose monomycolate exporter	
CAH1191574	-	-	
CAH1191578	-	-	
CAH1191582	-	-	
CAH1191586	-	-	
CAH1191590	-	-	
CAH1191594	rhaR_3	HTH-type transcriptional activator	
CAH1191598	-	-	
CAH1191602	-	-	
CAH1191606	-	-	
CAH1191611	mngB_1	Mannosylglycerate hydrolase	
CAH1191615	-	-	
CAH1191619	araQ_2	L-arabinose transport system permease	
CAH1191623	yteP_2	putative multiple-sugar transport system permease	
CAH1191626	rhaR_4	HTH-type transcriptional activator	
CAH1191629	glcR_1	HTH-type transcriptional repressor	
CAH1191632	-	-	
CAH1191635	RBKS	Ribokinase	
CAH1191638	iolG_1	Inositol 2-dehydrogenase/D-chiro-inositol 3-dehydrogenase	
CAH1191641	lsrF	3-hydroxy-5-phosphonooxypentane-2,4-dione thiolase	
CAH1192573	-	-	Non-ribosomal peptide synthetase cluster fragment
CAH1192575	araA_1	L-arabinose isomerase	
CAH1192577	rhaR_14	HTH-type transcriptional activator	
CAH1192579	iolE_2	Inosose dehydratase	
CAH1192581	-	-	
CAH1192583	-	-	
CAH1192585	yqfL_1	Putative pyruvate, phosphate dikinase regulatory protein	
CAH1192587	ppdK	Pyruvate, phosphate dikinase	
CAH1192589	-	-	
CAH1192591	yesR_1	Unsaturated rhamnogalacturonyl hydrolase	
CAH1192593	-	-	
CAH1192595	-	-	
CAH1192597	lgrD	Linear gramicidin synthase subunit D	
CAH1192599	mngB_5	Mannosylglycerate hydrolase	
CAH1192601	-	-	
CAH1192603	-	-	
CAH1192605	araQ_16	L-arabinose transport system permease	
CAH1192607	yteP_19	putative multiple-sugar transport system permease	
CAH1192608	-	-	
CAH1192610	rssB_3	Regulator of RpoS	
CAH1192612	-	-	
CAH1192614	lacZ_3	Beta-galactosidase	
CAH1192616	-	-	

Table S9. (cont.)

accession	gene	annotated product	type
CAH1200358	-	-	Proteusin cluster
CAH1200359	-	-	
CAH1200360	-	-	
CAH1200361	ohrR	Organic hydroperoxide resistance transcriptional regulator	
CAH1200362	-	-	
CAH1200363	araQ_28	L-arabinose transport system permease	
CAH1200364	yteP_30	putative multiple-sugar transport system permease	
CAH1200365	rhaR_35	HTH-type transcriptional activator	
CAH1200366	-	-	
CAH1200367	-	-	
CAH1200368	COQ5_2	2-methoxy-6-polyprenyl-1,4-benzoquinol methylase	
CAH1200369	rcsC_7	Sensor histidine kinase	
CAH1200370	codA_1	Cytosine deaminase	
CAH1200371	-	-	
CAH1200372	-	-	
CAH1200373	cmpD_1	Bicarbonate transport ATP-binding protein	
CAH1200374	mftE_1	Putative mycofactocin system creatinine amidohydrolase family protein	
CAH1200375	pnpB	p-benzoquinone reductase	
CAH1207745	gtfA	UDP-N-acetylglucosamine--peptide N-acetylglucosaminyltransferase GtfA subunit	Lasso peptide cluster
CAH1207749	-	-	Note: 100% similarity to BGC0001356, i.e. paeninodin
CAH1207753	-	-	
CAH1207755	-	-	
CAH1207759	glmU_1	Bifunctional protein	
CAH1207762	-	-	
CAH1207765	-	-	
CAH1207769	hepA_1	Heterocyst differentiation ATP-binding protein	
CAH1207774	-	-	
CAH1207778	-	-	
CAH1207782	-	-	
CAH1207786	-	-	
CAH1207790	-	-	
CAH1207793	asnO_1	Asparagine synthetase [glutamine-hydrolyzing] 3	
CAH1207796	-	-	
CAH1207800	-	-	
CAH1207804	-	-	
CAH1207808	-	-	
CAH1207812	-	-	
CAH1207816	-	-	
CAH1207822	-	-	
CAH1207827	-	-	
CAH1207832	yveL_1	Putative tyrosine-protein kinase	
CAH1207837	ywqC_2	putative capsular polysaccharide biosynthesis protein	
CAH1207842	-		

Table S9. (cont.)

accession	gene	annotated product	type
CAH1227117	-	-	Proteusin cluster
CAH1227119	-	-	
CAH1227122	-	-	Note: 60% similarity to BGC0001356, i.e. paeninodin
CAH1227126	hepA_2	Heterocyst differentiation ATP-binding protein	
CAH1227130	ettA_5	Energy-dependent translational throttle	
CAH1227134	-	-	
CAH1227138	-	-	
CAH1227140	-	-	
CAH1227144	-	-	
CAH1227149	-	-	
CAH1227153	-	-	
CAH1227157	rcsC_20	Sensor histidine kinase	
CAH1227161	-	-	
CAH1227165	-	-	
CAH1227169	-	-	
CAH1227173	-	-	
CAH1227177	-	-	
CAH1227181	-	-	
CAH1227185	rhaR_84	HTH-type transcriptional activator	
CAH1227189	yteP_95	putative multiple-sugar transport system permease	
CAH1228670	-	-	Non-ribosomal peptide synthetase cluster
CAH1228674	-	-	
CAH1228678	araQ_82	L-arabinose transport system permease	
CAH1228682	lacF_16	Lactose transport system permease	Note: 100% similarity to BGC000041 (i.e. paenibactin) and BGC0001185 (i.e. bacillibactin)
CAH1228686	dbpA_2	ATP-dependent RNA helicase	
CAH1228690	gerBA_16	Spore germination protein B1	
CAH1228694	gerBC_15	Spore germination protein B3	
CAH1228698	-	-	
CAH1228702	yndE_20	Spore germination	
CAH1228706	bceA_2	Bacitracin export ATP-binding protein	
CAH1228710	yxdM	ABC transporter permease	
CAH1228714	graS_2	Sensor histidine kinase	
CAH1228718	graR_2	Response regulator	
CAH1228722	-	-	
CAH1228726	-	-	
CAH1228730	-	-	
CAH1228734	hsrA_2	putative transport protein	
CAH1228739	mbtH	-	
CAH1228743	dhbF	Dimodular nonribosomal peptide synthase	
CAH1228747	dhbB	Isochorismatase	
CAH1228752	dhbE	2,3-dihydroxybenzoate-AMP ligase	
CAH1228757	dhbC	Isochorismate synthase	
CAH1228761	dhbA	2,3-dihydro-2,3-dihydroxybenzoate dehydrogenase	
CAH1228765	besA	Ferri-bacillibactin esterase	
CAH1228769	-	-	
CAH1228773	feuC_1	Iron-uptake system permease	
CAH1228777	feuB_1	Iron-uptake system permease	
CAH1228781	feuA	Iron-uptake system-binding protein	
CAH1228785	btr_10	HTH-type transcriptional activator	
CAH1228789	-	-	
CAH1228793	-	-	
CAH1228797	-	-	
CAH1228801	-	-	
CAH1228805	-	-	
CAH1228809	-	-	
CAH1228813	-	-	
CAH1228817	-	-	

Table S9. (cont.)

accession	gene	annotated product	type
CAH1230861	COQ3_4	Ubiquinone biosynthesis O-methyltransferase	Terpene
CAH1230864	sasA_25	Adaptive-response sensory-kinase	
CAH1230867	walR_8	Transcriptional regulatory protein	
CAH1230868	sufS_3	Cysteine desulfurase	
CAH1230871	graR_3	Response regulator	
CAH1230874	graS_3	Sensor histidine kinase	
CAH1230877	bceA_3	Bacitracin export ATP-binding protein	
CAH1230881	bceB_2	Bacitracin export permease	
CAH1230885	-	-	
CAH1230889	-	-	
CAH1230891	-	-	
CAH1230895	ydbD_2	putative manganese catalase	
CAH1230899	-	-	
CAH1230902	-	-	
CAH1230906	mccF	Microcin C7 self-immunity protein	
CAH1230910	-	-	
CAH1230914	fabG_11	3-oxoacyl-[acyl-carrier-protein] reductase	
CAH1230918	-	-	
CAH1230924	-	-	
CAH1230928	-	-	

Table S10. Cellular fatty acid profiles of strain JJ-42^T and type strains of closely related species of the genus *Paenibacillus*

Strains: 1,JJ-42^T; 2, *Paenibacillus pectinilyticus* KCTC 13222^T; 3, *Paenibacillus qinlingensi* TEGT-2^T; 4, *Paenibacillus frigoriresistens* CCTCC AB 2011150^T; 5, *Paenibacillus ferrarius* CCTCC AB 2013369^T; 6, *Paenibacillus alginolyticus* NBRC 15375^T; 7, *Paenibacillus chondroitinus* DSM 5051^T. Data for taxon 1 and 3 from this study. All other data are from Xin et al. (2017); the six strains were cultured on R2A Agar for 2 days at 28 °C and cells from the third streak quadrant of the agar plates were used. Values are percentages of the total fatty acids; -, not detected

**fatty acid**	**1**	**2**	**3**	**4**	**5**	**6**	**7**
iso-$C_{14:0}$	2.9	1.8	2.9 (3.8)	2.6	3.1	2.0	3.7
$C_{14:0}$	-	2.6	- (1.9)	0.9	2.8	0.6	1.3
iso-$C_{15:0}$	6.1	3.6	4.3 (4.0)	8.1	5.5	6.2	8.7
anteiso-$C_{15:0}$	69.3	62.5	75.8 (70.9)	66.8	64.3	69.2	62.2
$C_{15:0}$	2.7	2.6	1.9 (0.8)	0.8	1.1	-	0.7
iso-$C_{16:0}$	10.4	9.4	8.5 (10.2)	9.9	11.1	10.6	12.3
$C_{16:1}\omega 11c$	-	-	- (-)	0.3	-	-	-
$C_{16:0}$	4.4	8.1	2.8 (4.9)	4.2	5.1	4.5	6.4
iso-$C_{17:0}$	-	0.7	- (0.3)	1.9	2.2	1.6	1.2
anteiso-$C_{17:0}$	4.2	5.8	3.7 (3.2)	4.0	4.8	5.3	3.5
$C_{18:1}\omega 9c$	-	1.7	-	0.5	-	-	-
Summed feature 8*	-	1.2	-	-	-	-	-

*Summed feature 8 comprises $C_{18:1}\omega 7c$ and/or $C_{18:0}\omega 6c$

Figure S1. Polar lipid profile of strain JJ-42^T after staining with 5% ethanolic molybdatophosphoric acid and development at 140 °C.

Abbreviations: DPG, diphosphatidylglycerol; PG, phosphatidylglycerol; PE, phosphatidylethanolamine; APL1-3, unidentified aminophospholipids; L, unidentified polar lipid.

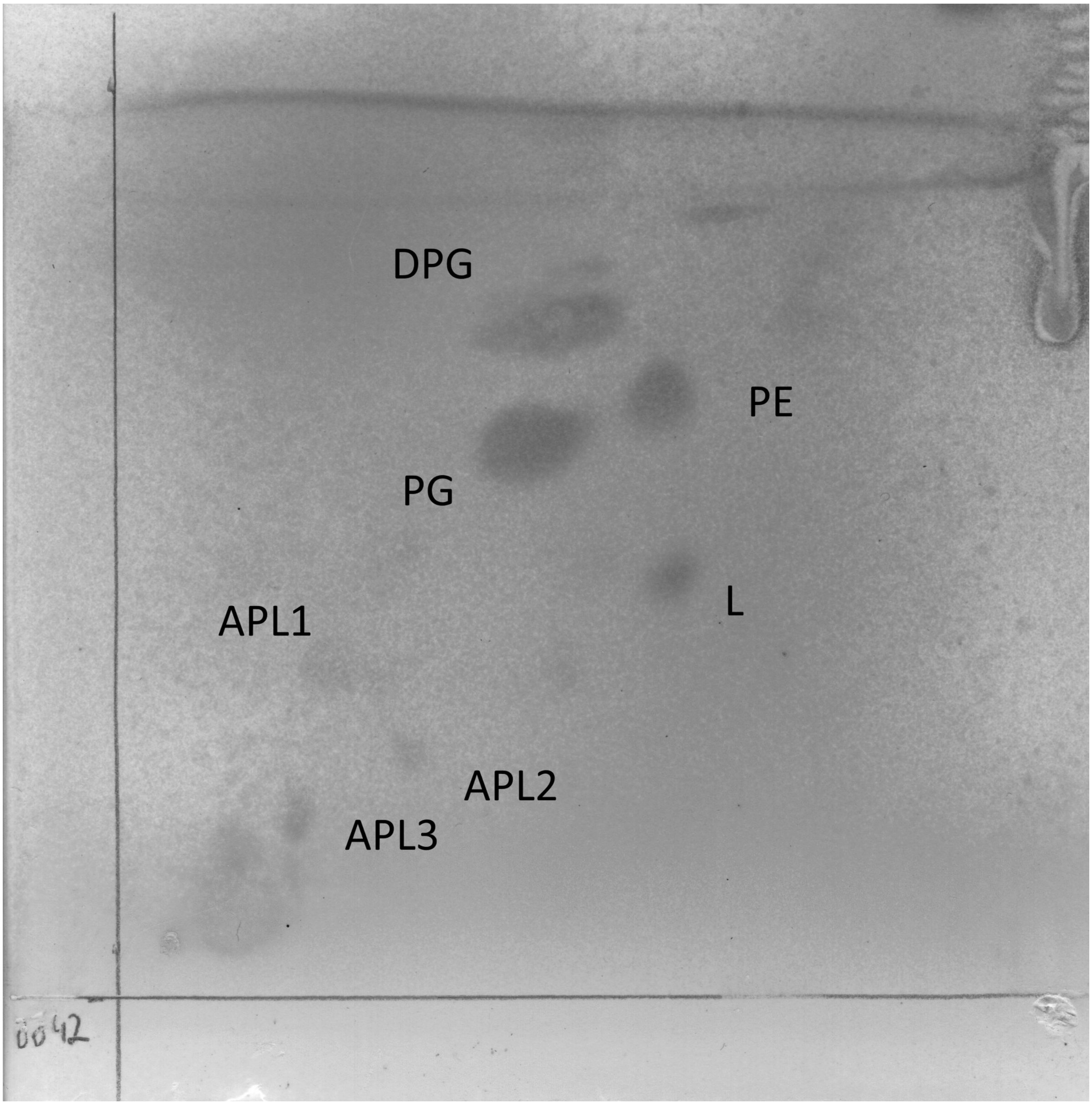

Supplement: Supplementary file 1 — Supplementary file1 (PDF 660 KB) [file 203_2022_3225_MOESM1_ESM.pdf]
